# Understanding optical reflectance contrast for real‐time characterization of epithelial precursor lesions

**DOI:** 10.1002/btm2.10137

**Published:** 2019-06-27

**Authors:** Si Chen, Xin Ge, Xinyu Liu, Qianshan Ding, Nanshuo Wang, Xianghong Wang, Shufen Chen, Haitao Liang, Yunchao Deng, Qiaozhou Xiong, Guangming Ni, En Bo, Chenjie Xu, Honggang Yu, Linbo Liu

**Affiliations:** ^1^ School of Electrical and Electronic Engineering Nanyang Technological University Singapore Singapore; ^2^ Department of Gastroenterology Renmin Hospital of Wuhan University Wuhan China; ^3^ State Key Laboratory of Electronic Thin Films and Integrated Devices, School of Optoelectronic Information University of Electronic Science and Technology of China Chengdu China; ^4^ School of Chemical and Biomedical Engineering Nanyang Technological University Singapore Singapore

**Keywords:** epithelial cancer, keratin filament, mucin granule, optical biopsy, optical coherence tomography, optical reflectance contrast, precancerous lesion

## Abstract

Detecting early‐stage epithelial cancers and their precursor lesions are challenging as lesions could be subtle and focally or heterogeneously distributed over large mucosal areas. Optical coherence tomography (OCT) that enables wide‐field imaging of subsurface microstructures in vivo is a promising screening tool for epithelial diseases. However, its diagnostic capability has not been fully appreciated since the optical reflectance contrast is poorly understood. We investigated the back‐scattered intensities from clustered or packed nanometer scale intracellular scatterers using finite‐difference time‐domain method and 1‐μm resolution form of OCT, and uncovered that there existed correlations between the reflectance contrasts and the ultrastructural clustering or packing states of these scatterers, which allows us to interpret the physiological state of the cells. Specifically, both polarized goblet cells and foveolar cells exhibited asymmetric reflectance contrast, but they could be differentiated by the optical intensity of the mucin cup due to the different ultrastructural make‐ups of the mucin granules; keratinocytes could demonstrate varied cytoplasmic intensity and their cytoplasmic contrast was closely correlated with the packing state of keratin filaments. Further preliminary study demonstrated that these new understandings of OCT image contrast enables the characterization of precancerous lesions, which could complement the current morphology‐based criteria in realizing “virtual histology” and would have a profound impact for the screening and surveillance of epithelial cancers.

## 1 INTRODUCTION

Epithelial cancers rank in the leading causes of mortality worldwide.^1^, ^2^, ^3^ Although detecting cancers at their early or precancerous stages is associated with favorable prognosis, it remains a major challenge in routine clinical practice. In most cases, these early‐stage lesions are difficult to be recognized by the eye^4^, ^5^, ^6^, ^7^ and can be focally and heterogeneously distributed over a large mucosal area.^5^, ^6^, ^8^, ^9^ Therefore, biopsies often have to be randomly performed on multiple portions of the mucosa, with the hope of sampling changes of particular clinical relevance, which unfortunately is frequently missed.^5^, ^6^, ^10^ Histological analysis of stained, thin sections from resected materials remains the gold standard for a definitive diagnosis, where contrast agents are adopted to specifically highlight structures of interest with clear understandings on the underlying mechanistic chemistry of staining. However, this workup is time‐consuming and labor‐intensive which limits the capability of clinicians to immediately characterize the lesions, possibly leading to unnecessary biopsies or the need for repeated biopsies. In addition, concerns on the costs from the histological assessment have been raised when lesions of limited clinical importance are increasingly found as is the case in diminutive colorectal polyps.^11^, ^12^

Optical imaging modalities have attracted significant interests for “virtual histology” where resolving cellular or subcellular information in situ is possible without the need of histological assessment. Confocal fluorescence endomicroscopy provides satisfactory cellular details by use of contrast agents that could selectively stain the nucleus, cytoplasm, or extracellular matrix.^13^, ^14^ However, the limited field of view (~ 400 × 400 μm) makes it difficult for wide‐field imaging.^13^, ^14^ Optical coherence tomography (OCT) is capable of imaging subsurface microstructures at cellular resolution across a large mucosal area.^15^, ^16^, ^17^, ^18^ The reflectance (back‐scattering) contrast is provided by the refractive index differences between microstructures. Whereas, unlike histological methods where the image contrasts have been fully understood and well matched with the physiological events in tissues, optical‐reflectance image interpretation has ever relied on morphological or architectural similarities to histology, leaving the biological bases underlying the contrast poorly understood.^16^, ^17^, ^19^, ^20^, ^21^ This knowledge gap has relegated these modalities to nonspecific morphometric tools where reflectance signals that may carry diagnostically important information are missed, precluding their applications in a broader research or clinical arena.

Epithelial cells are typically specialized with variations in their intracellular inclusions, suited to the particular task in a specific organ.^22^ The light scattering from cells has been investigated both experimentally and theoretically. While cell nuclei have a quasi‐uniform distribution of its compositions and consistently present low back‐scattering in the core,^21^, ^23^, ^24^ the cytoplasm is the predominant site reflecting the specialization‐mediated intracellular variations and corresponding alterations in their back‐scattered intensity. Chen et al. measured the back‐scattered intensity of the nuclear cores and cytoplasm in the stratified squamous epithelium to be in the order of 10^−7^ and 10^−6^, respectively, when they are normalized to a perfect reflector.^23^ Saidi et al. used Mie theory to approximate skin tissue scattering but by assuming cells are homogeneous spheres of a single size.^25^ Dunn and Drezek et al. used the finite‐difference time‐domain (FDTD) method to model light scattering from cells containing multiple organelles of arbitrary shape.^26^, ^27^ Their studies suggest that small organelles whose size is comparable to the wavelength of light play a more important role than the nucleus in scattering from a cell,^26^, ^27^ such as mitochondria^28^ and melanin granules.^29^

However, most of the previous efforts were focused on the scattering contributions from micrometer‐scale quasi‐spherical scatterers, and little is understood on the back‐scattering (reflectance) from nanometer scale scatterers, in particular, clustered mucin granules and packed keratin filaments and microvilli. Furthermore, the effects of their biological variations on the back‐scattered intensity of cells under different physiological states are largely unknown. In this study, we investigated the micro‐ and ultra‐structural bases underlying the cytoplasmic optical reflectance contrast in a wide variety of specialized epithelia both theoretically using the FDTD method and experimentally using a cellular‐resolution OCT (μOCT). We further validate the feasibility of the improved understandings on the optical reflectance contrast of epithelial cells for real‐time characterization of epithelial cancers and their precursors.

## 2 RESULTS

We firstly developed back‐scattering models of mucin granules (Supporting Information Figure 1), microvilli (Supporting Information Figure 2), and keratin filaments (Supporting Information Figure 3) using the FDTD method according to transmission electron microscopic (TEM) images and previously published data (Table 1). The results show that these clustered or packed nanometer‐scale scatterers may contribute significantly or even dominantly to cell back‐scattering, depending on their clustering or packing states. The corresponding experimental realizations were carried out using μOCT on intact mammalian epithelia including simple columnar epithelia from swine stomach, colon and small intestine, and stratified squamous epithelia (SSE) from swine skin (orthokeratinized), esophagus (parakeratinized), and floor of month (nonkeratinized), respectively.^23^ On one hand, we validated our theoretical predictions by correlating the cytoplasmic reflectance contrasts with the micro‐ or ultra‐structural features of the above mentioned cytoplasmic inclusions pertaining to the physiological states of specialized epithelial cells; on the other, our theoretical analysis provide meaningful explanations of the mechanisms underlying the observed back‐scattering phenomena. Thereafter, we conducted a preliminary study to evaluate the feasibility of the new understandings on the image contrast for the interpretation of back‐scattering features in precancerous lesions using specimens from the mice esophageal and human gastrointestinal tracts.

**Table 1:** Parameters of cytoplasmic scatterers in FDTD simulation

Scatterer	Biological variation	Shape	Diameter	Length	Spacing	Refractive index
Mucin granule	Foveolar cell	Sphere	202 nm	–	380 nm	1.38–1.41^30^
	Goblet cell	Sphere	1,230 nm	–	0	–
Microvillus	Enterocyte	Cylinder	100 nm^36^	1,085 nm^36^	93.5 nm^36^	1.38–1.41^30^
	Colonocyte	Cylinder	90 nm	140–510 nm	140–340 nm	1.38–1.41^30^
Keratin filament	Keratinocyte (closely packed)	Cylinder	8 nm	500 nm	16 nm	1.40
	Keratinocyte (loosely dispersed)	Cylinder	8 nm	500 nm	24 nm	1.40

## 3 CYTOPLASMIC OPTICAL REFLECTANCE CONTRAST OF NORMAL EPITHELIUM

### 3.1 Simple columnar epithelia

**Figure 1:**
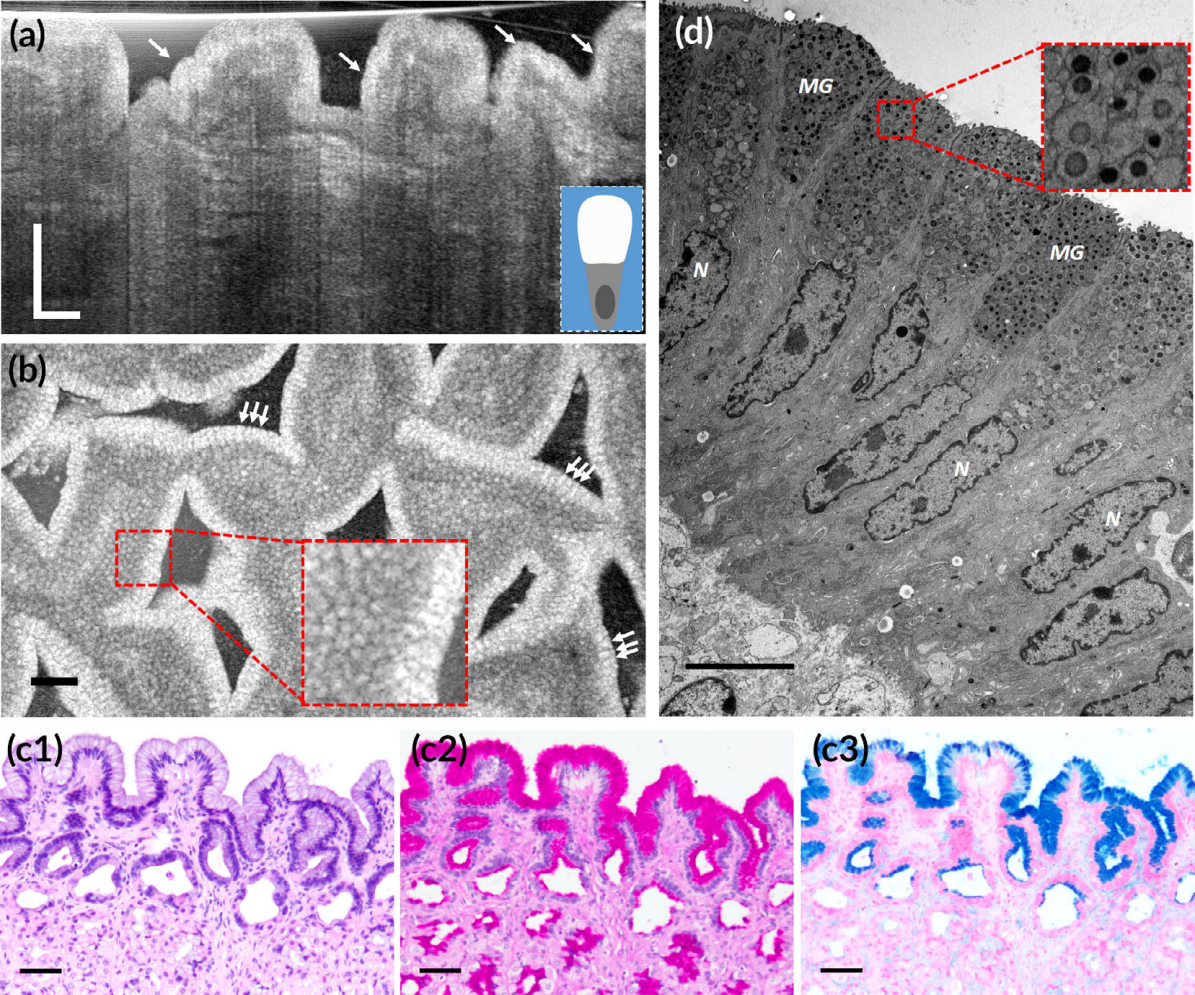
Subcellular reflectance contrast of swine gastric mucosa. (a and b) Representative μOCT cross‐sectional (a) and en face (b) images acquired ex vivo present bright apical portion of foveolar cells (white arrows); the inset in (a) is a schematic of the back‐scattering profile of a foveolar cell; the red dashed box in (b) is zoomed‐in by 3×. (c1–c3) Corresponding histology with H&E, PAS, and AB staining, respectively; (d) TEM image at a magnification of 5,000× and the red dashed box is zoomed‐in by 4× showing mucin granules (MGs) of foveolar cells. N, nucleus. Scale bars for μOCT and routine histological images, 50 μm; scale bars for TEMs, 5 μm

**Figure 2:**
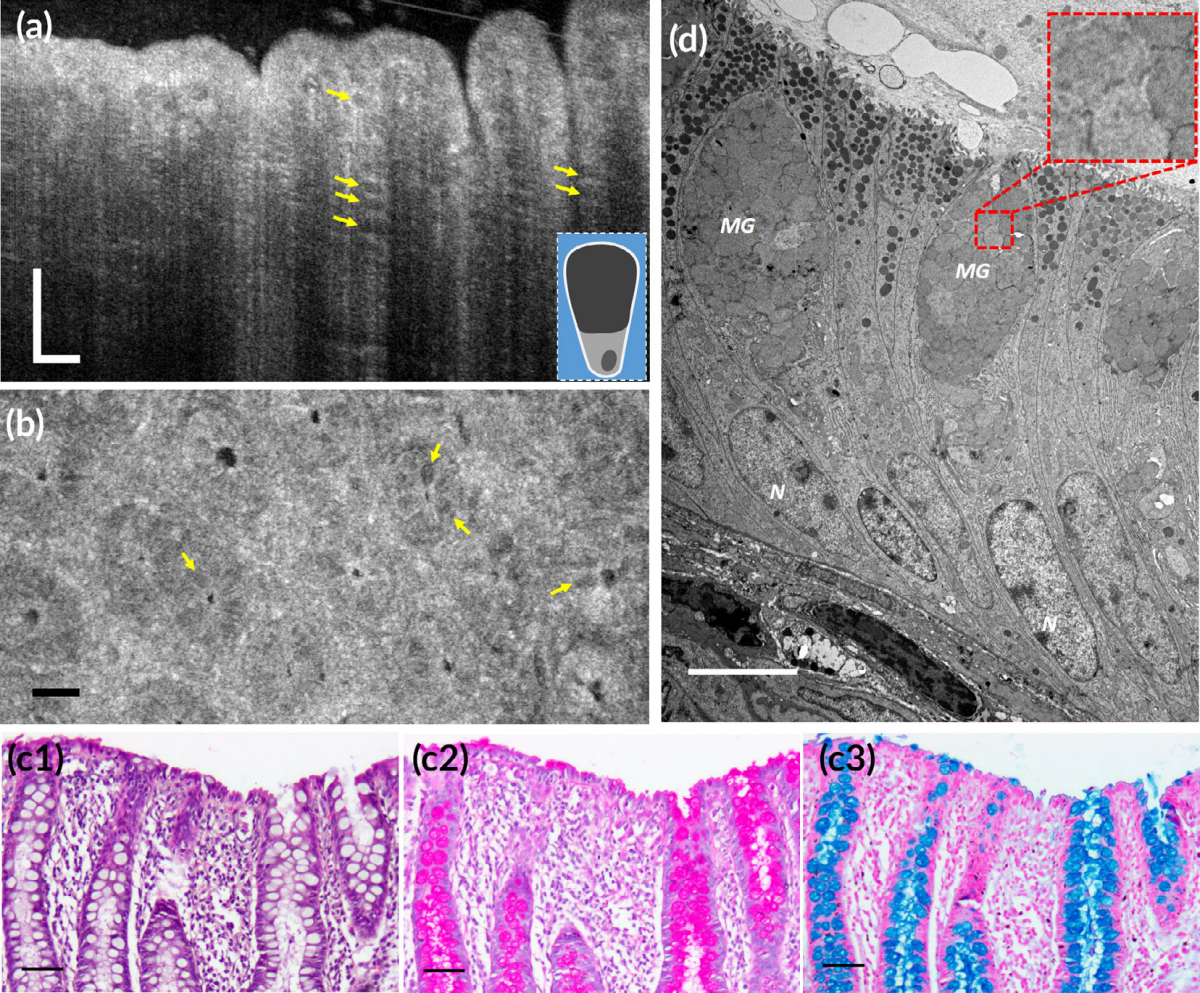
Subcellular reflectance contrast of swine colonic mucosa. (a and b) Representative μOCT cross‐sectional (a) and en face (b) images obtained ex vivo demonstrate low‐reflectance apical portion of goblet cells (yellow arrows); the inset in (a) is a schematic of the back‐scattering profile of a goblet cell. (c1–c3) Corresponding histology with H&E, PAS, and AB staining, respectively; (d) TEM image at a magnification of 5,000× and the red dashed box is zoomed‐in by 4× showing mucin granules (MGs) of goblet cells. N, nucleus. Scale bars for μOCT and routine histological images, 50 μm; scale bars for TEMs, 5 μm

**Figure 3:**
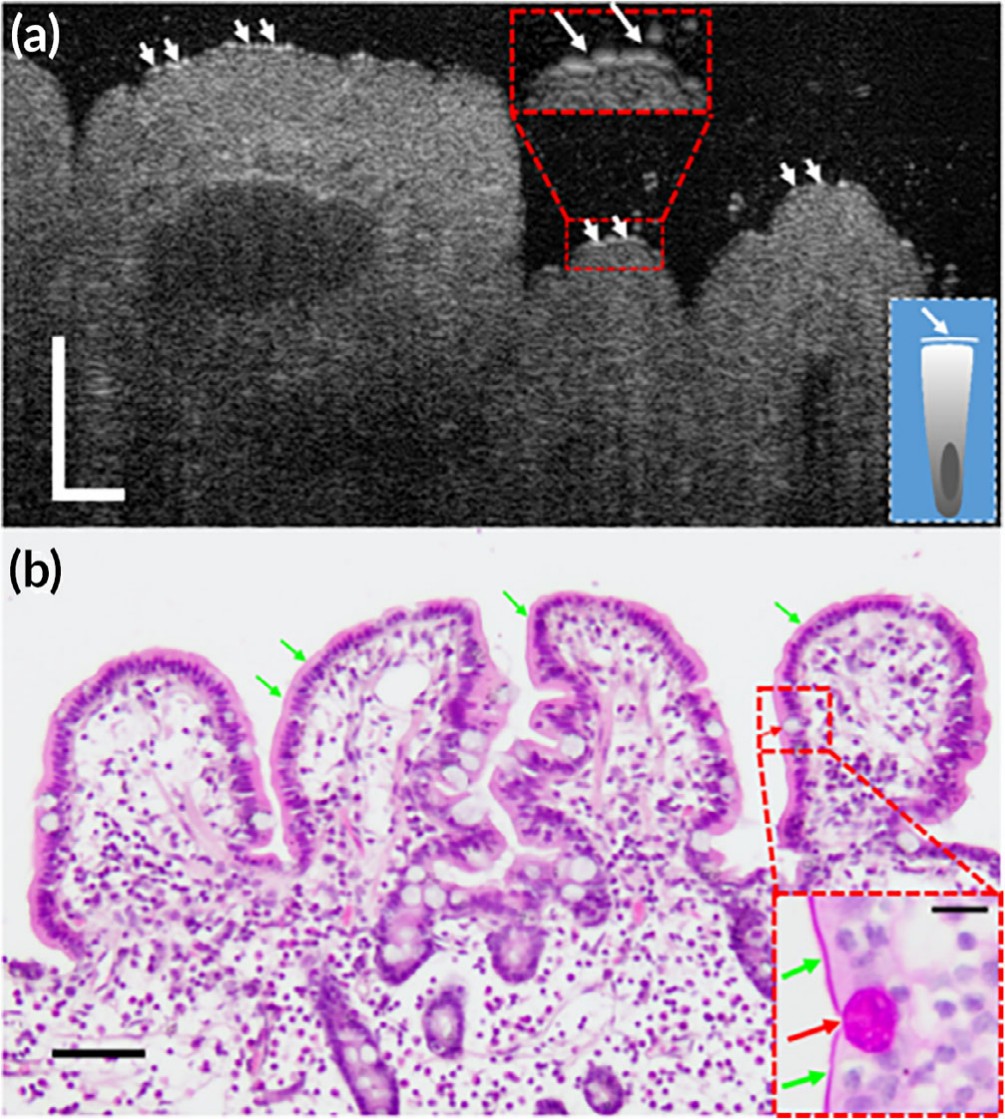
Subcellular reflectance contrast of swine small intestine. (a) A representative μOCT cross‐sectional image acquired in vivo showing a high‐scattering fine structure overlying the apical surface of enterocytes (white arrows), suggestive of brush border. The lower‐right inset is a schematic of the scattering profile of an enterocyte. (b) Corresponding histology with H&E staining and the red dashed box is zoomed in by 3× with PAS staining that highlights brush border (green arrows) and goblet cell (red arrows). Scale bars for original images, 50 μm; scale bar for inset in (b), 10 μm

Previous studies suggest that gastric foveolar cells^20^, ^32^, ^33^, ^34^ and intestinal goblet cells^34^, ^35^ present significant reflectance contrasts in OCT images, so we focused our studies on these two cell types (Figures 1 and 2). We additionally investigated enterocytes in the small intestine (Figure 3) due to the new finding of brush border reflection.

#### 3.1.1 Reflectance contrasts of polarized mucin‐secreting cells

Both foveolar cells and goblet cells are highly polarized mucin‐secreting cells which are important components of the gastrointestinal tract by producing the uttermost defense line of the epithelia. Although both cells demonstrated a similar “empty” appearance of the apical cytoplasm in H&E stained sections and have the same histochemical properties of mucins (Figures 1c1–c3 and 2c1–c3), their ultrastructural make‐ups are very different: the mucin cups of foveolar cells were heterogeneously filled with nanometer‐scale, discrete, electron‐dense granules circled by relatively low‐electron‐density surroundings; in contrast, those of goblet cells were homogenous and full of fused, low‐electron‐density mucin granules (Figures 1d and 2d). We modeled mucin granules in foveolar cells as nanoscale spheres suspended in the cytosol. The refractive index data of mucin granules is not available so the refractive index range of cell organelles was used as the best estimate (Table 1).^30^ The simulated normalized back‐scattered intensity ranges from (6.7–18) × 10^−6^, which is one of the brightest intracellular structures investigated in this study (Supporting Information Table 1). We predicted that the back‐scattering intensity from mucin granules of goblet cells were insignificant since there was no noticeable electron density change in the TEM images and thus simulation was not conducted.

In μOCT images, the asymmetric optical contrasts of mucin‐secreting cells well reproduced the polarization feature of the cells (Figures 1a and 2a). In agreement with our simulation results, foveolar cells demonstrated a high reflectance contrast in the apical cytoplasm relative to the low intensity in the basal portion where nuclei resided (Figure 1a,b, white arrows). These bright signals lining gastric mucosa signified well‐polarized foveolar cells, a finding that has unfortunately long been overlooked^20^, ^32^ or misunderstood.^33^, ^34^ In contrast, goblet cells could be identified by the low reflectance contrast of their barrel‐shaped apical cytoplasm relative to adjacent enterocytes whose apical portion was relatively bright (Figure 2a,b, yellow arrows). These back‐scattering features of goblet cells agreed with previous reports in human and rat.^34^, ^35^ The normalized back‐scattered intensity of the mucin cups of foveolar cells was measured to be (2.74 ± 0.09) × 10^−6^, which was significantly higher than that of goblet cells (0.28 ± 0.02) × 10^−6^ (Figure 8a; independent‐samples *t* test; *p* < .01), which agrees well with their ultrastructural differences (Figures 1d and 2d) and the theoretical predictions.

#### 3.1.2 Reflectance contrast of brush border

Enterocytes are epithelial cells fulfilling the function of absorption, whose apical surfaces are characterized by the presence of microvilli driven by polarization. The tips of microvilli are tightly and evenly packed, and the surface formed by their tips is equivalent to a high grade optical surface (Supporting Information Table 2). We modeled microvilli as nanocylinders and used the previously reported refractive index range of cell organelles as the best estimate (Table 1).^30^ FDTD simulation results show that the normalized back‐scattered intensity of brush border ([2.5–7.4] × 10^−5^) was at least one order of magnitude higher than most of the cytoplasmic components in this study (Supporting Information Tables 1–3 and Figure 8). According to this prediction, a bright line in μOCT images at the epithelial surface of the small intestine was expected. We also simulated microvilli in the colon epithelium, and the results show that the decrease in the packing density leads to a dramatic decrease in the back‐scattered intensity ([3.9–12] × 10^−6^; Supporting Information Table 2).

In μOCT images of the small intestine, we did identify high‐reflectance contour‐like signals lining the luminal epithelial cell surface in vivo (Figure 3a, white arrows), which could seldom be observed in the stomach and colon. The height of the structure was1.35 ± 0.07 μm away from the epithelial surface in μOCT images (refractive index = 1.33; *n* = 20), similar to that in histology (1.23 ± 0.04 μm; *n* = 20). The measured normalized back‐scattered intensity was (1.26 ± 0.10) × 10^−5^ (*n* = 100) agree well with the simulation result. Such a strong back‐scattered signal can only be from the brush border (Figure 3b**,** inset, green arrows) and the measured value.

### 3.2 Stratified squamous epithelia

**Figure 4:**
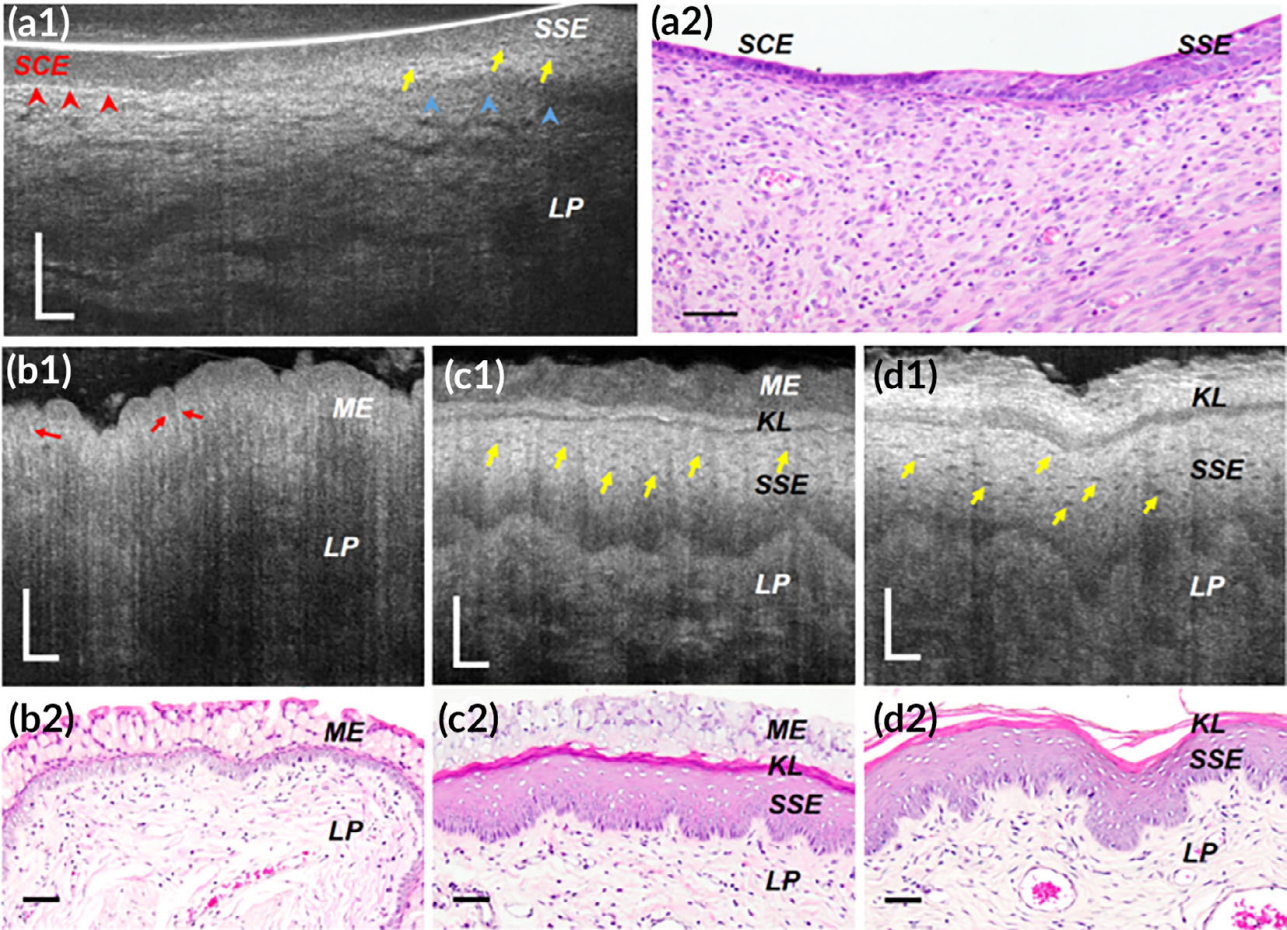
Subcellular reflectance contrast of albino rat cervix in vivo (a1 and a2) and vagina ex vivo (b1–d2). (a1, b1, c1, and d1) Representative μOCT cross‐sectional images and (a2, b2, c2, and d2) corresponding histology with H&E staining. (a1 and a2) Cervical epithelium at squamocolumnar junction showed low‐scattering simple columnar epithelium (SCE; red arrowheads) and high‐scattering stratified squamous epithelium (SSE; blue arrowheads). (b1 and b2) At diestrus, vaginal epithelial cells were full of mucin granules demonstrating middle‐low optical intensity. (c1 and c2) As it approached proestrus, mucification gave way to keratinization: Keratinocytes manifested high cytoplasmic intensity, with respect to the relatively low‐scattering mucified cells at the epithelial surface. (d1 and d2) With the estrogen surge at estrus, vaginal epithelial keratinization reached its maximum, characterized by a well‐developed keratinized layer and keratinocytes present bright cytoplasm and readily recognizable nucleocytoplasmic contrast. All yellow arrows represent dark nuclei and red arrows indicate bright cell borders. KL, keratinized layer; LP, lamina propria; ME, mucified epithelium. Scale bars, 50 μm

In μOCT images of the squamocolumnar junction of albino rat cervix in vivo, keratinocytes presented high‐scattering cytoplasm, in striking contrast with the adjacent low‐scattering columnar cells (Figure 4a1,a2). These observations indicated that the high cytoplasmic intensity was correlated with keratinization, which could also be supported by the evidences from albino rat vagina ex vivo whose epithelium could alternate between mucification and keratinization provoked by progesterone and estrogen respectively during estrous cycle (Figure 4b1–d2).

**Figure 5:**
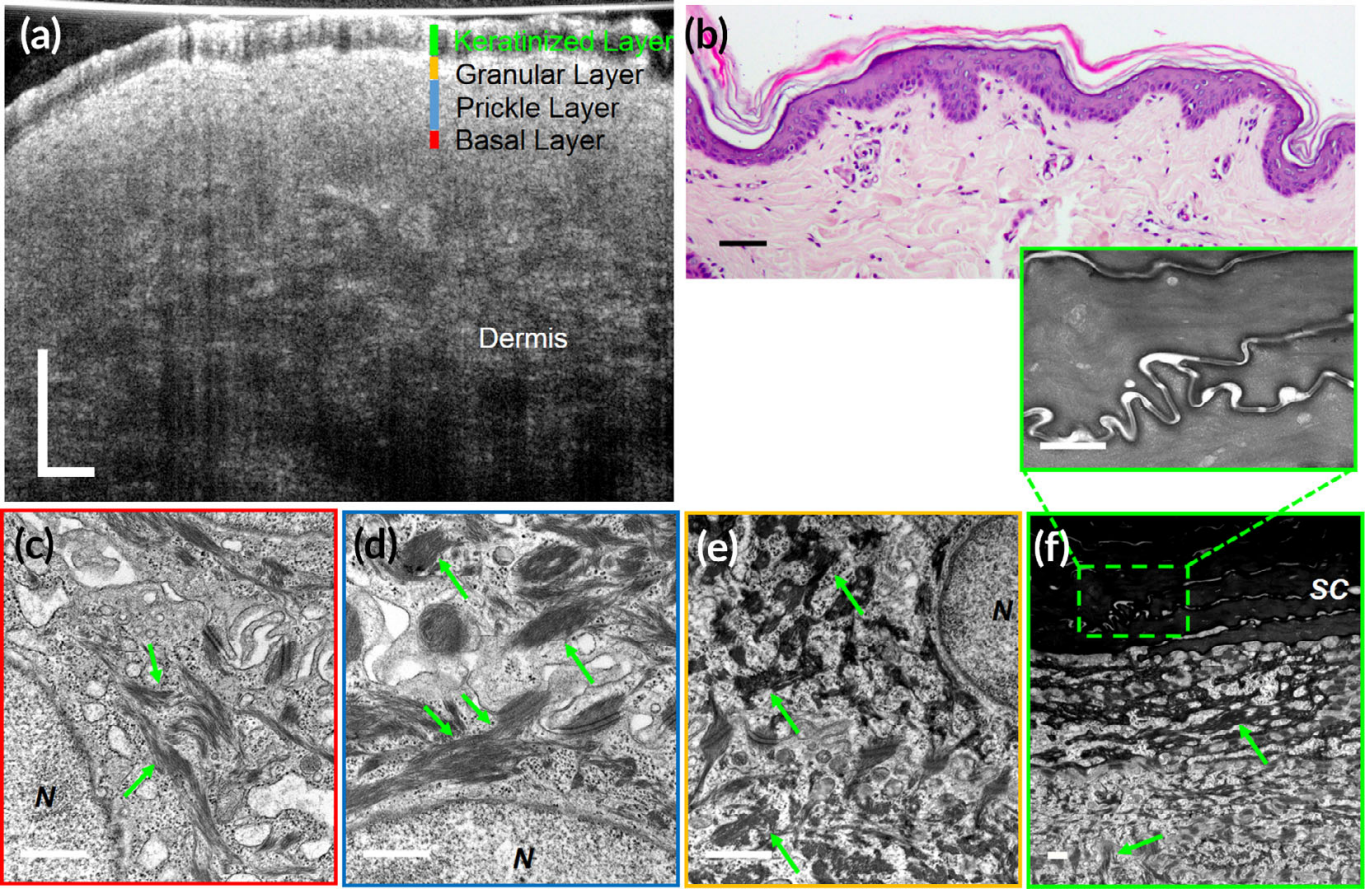
Subcellular reflectance contrast of keratinocytes in swine epidermis (orthokeratinized epithelium). (a) A representative in vivo μOCT cross‐sectional image; (b) corresponding histology with H&E staining; (c–f) representative TEMs of cells from the basal layer (c), prickle layer (d), granular layer (e), and keratinized layer (zoomed‐in view of rectangular area in f) at 50,000× magnification, respectively. All green arrows indicate keratin filament bundles. N, nucleus; SC, stratum corneum. Scale bars for μOCT and H&E stained images, 50 μm; scale bars for TEMs, 0.5 μm

**Figure 6:**
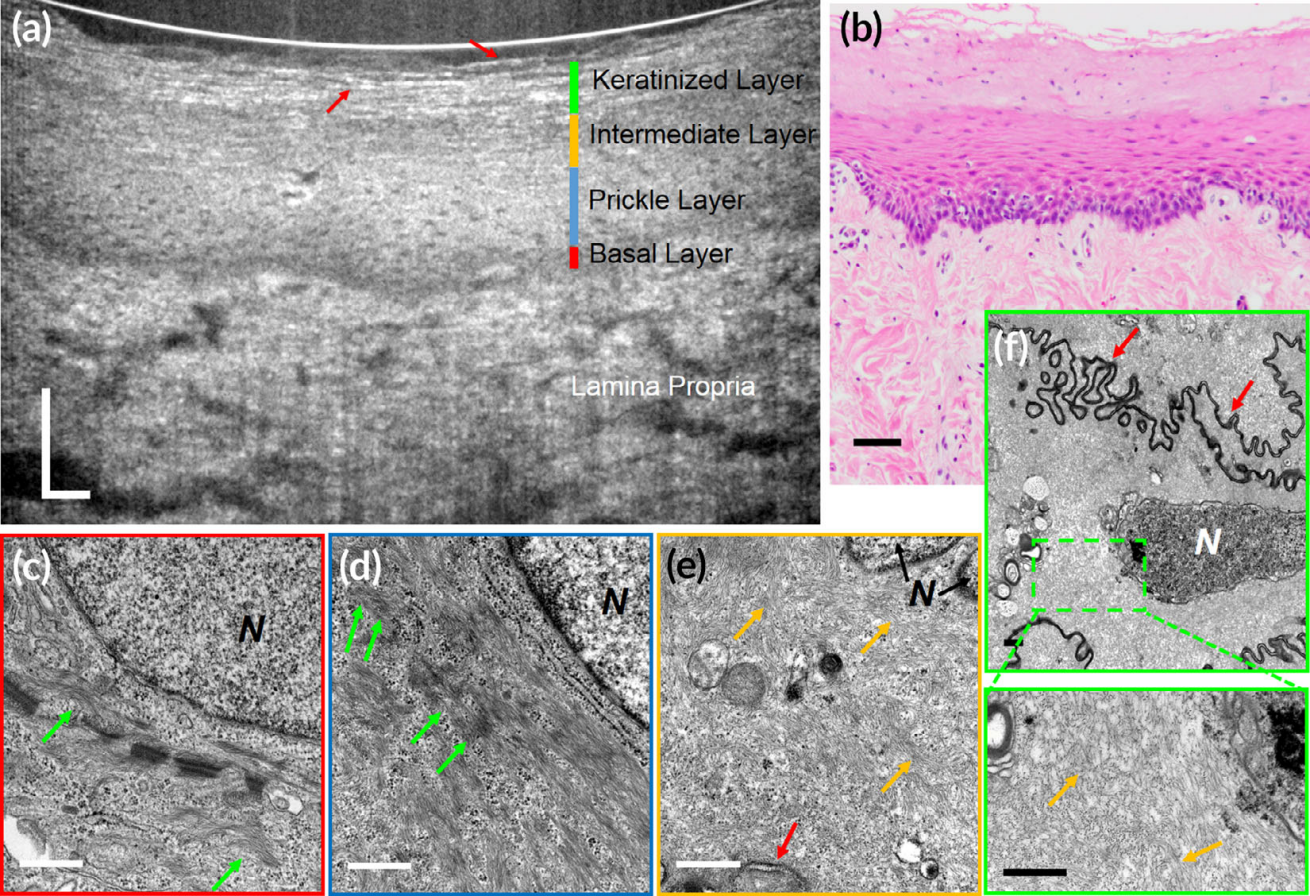
Subcellular reflectance contrast of keratinocytes in swine esophagus (parakeratinized epithelium). (a) A representative in vivo μOCT cross‐sectional image; (b) corresponding histology with H&E staining; (c–f) representative TEMs of cells from the basal layer (c), prickle layer (d), intermediate layer (e), and keratinized layer (zoomed‐in view of rectangular area in f) at 50,000× magnification, respectively. Green and orange arrows indicate keratin filament bundles and individual keratin filament, respectively; red arrows indicate cell borders. N, nucleus. Scale bars for μOCT and H&E stained images, 50 μm; scale bars for TEMs, 0.5 μm

**Figure 7:**
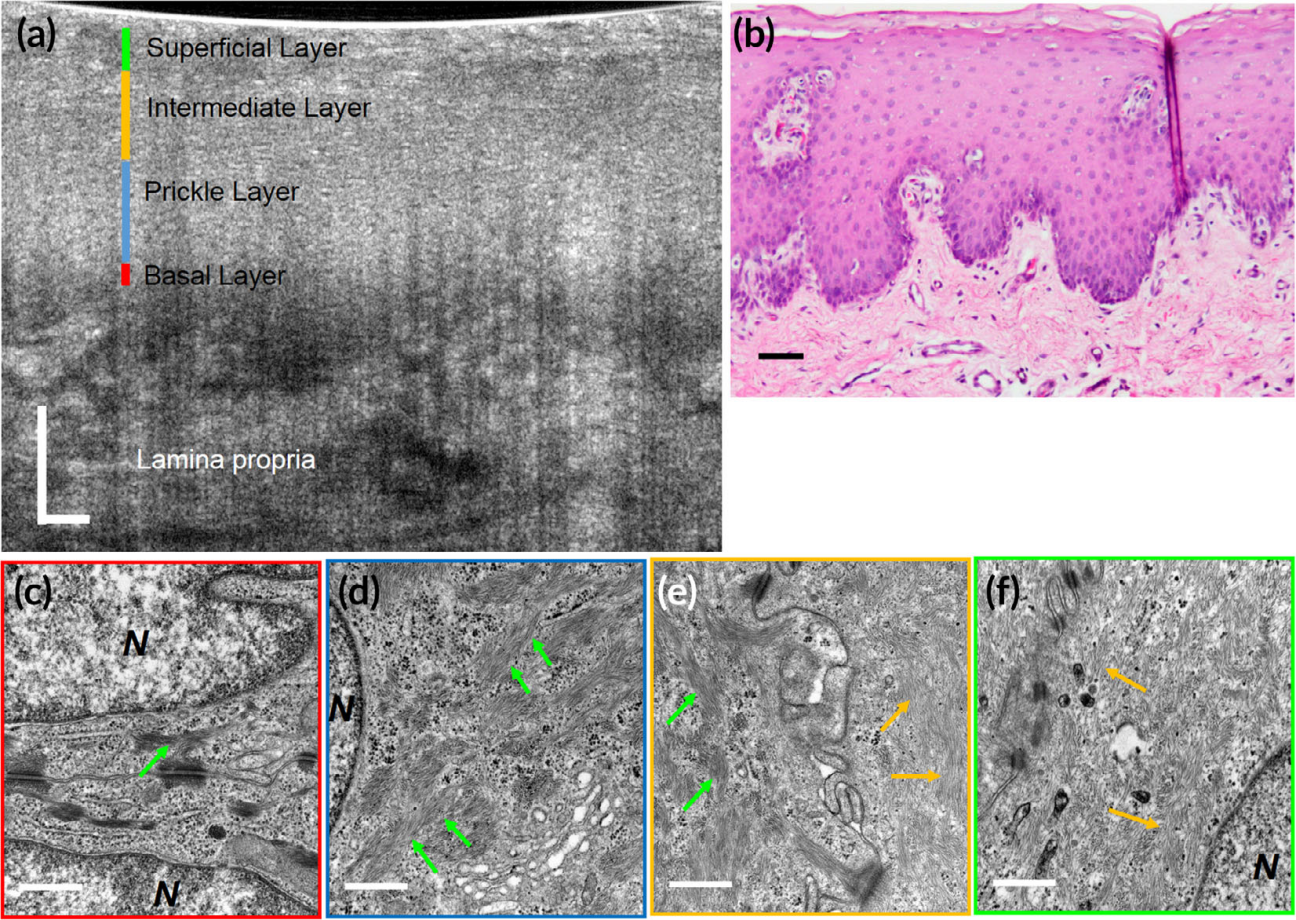
Subcellular reflectance contrast of keratinocytes in swine floor of month (nonkeratinized epithelium). (a) A representative ex vivo μOCT cross‐sectional image; (b) corresponding histology with H&E staining; (c–f) representative TEMs of cells from the basal layer (c), prickle layer (d), transition between prickle and intermediate layer (e), and superficial layer (f) at 50,000× magnification, respectively. Green and orange arrows indicate tonofilament bundles and individual tonofilament, respectively. N, nucleus. Scale bars for μOCT and H&E stained images, 50 μm; scale bars for TEMs, 0.5 μm

At the ultrastructural level, the clustering or packing states of keratin filaments vary dramatically between keratinocytes of different degree of keratinization (Figures 5, 6, 7). Generally, two types of packing states could be observed: (a) closely packed and well aligned keratin filaments in highly clustered bundles, which are also known as tonofibrils, such as those in the prickle layer and the granular layer of the orthokeratinized epidermis (Figure 5d,e), and the prickle layer of the parakeratinized esophageal epithelium (Figure 6d) and nonkeratinized oral mucosa (Figure 7d); (b) Much less aggregated, loosely dispersed individual filament such as those in the intermediate layers of the parakeratinized esophageal epithelium (Figure 6e), and nonkeratinized oral mucosa (Figure 7e**,** yellow arrows) in swine. In particular, in the swine esophageal epithelium, we could see clearly the variation of the packing state as the cell evolved from the prickle layer to the intermediate layer (Figure 6d and e). We developed FDTD models of the two packing states of keratin filaments (Supporting Information Figure 3), and the results show that the normalized backscattered intensity of tonofilaments in closely packed state was 5.46 × 10^−6^, which was larger than those in the loosely dispersed state 3.08 × 10^−6^ (Supporting Information Table 3).

In μOCT images of swine SSEs with different degree of keratinization, we found that the cytoplasmic optical intensity of keratinocytes was indeed associated with the packing states of intracellular keratin filaments: cells presenting bright cytoplasm had filaments that were closely packed into bundles; those demonstrating relatively low intensity contained filaments that were loosely dispersed within cytoplasm (Figures 5, 6, 7). Note that the basal layer of nonpigmented SSEs sometimes seemed to be low scattering although filaments in basal cells were also packed into electron dense bundles (Figures 5c, 6c**,** and 7c). It is possibly owing to the fact that these cells have large nucleocytoplasmic ratio and are generally crowded to each other so that the scattering feature at this region is dominated by that of nuclei. Besides, imaging artifacts such as light attenuation may also contribute to its low optical intensity relative to the overlying epithelial layers. Also, the keratinized layer in orthokeratinized SSE could demonstrate either high intensity or low intensity (Figure 5a). The low intensity was possibly due to the fact that these terminally keratinized cells were highly dehydrated, compact, and filled only with keratin filaments, rendering a homogenous distribution of refractive index (Figure 5f).

**Figure 8:**
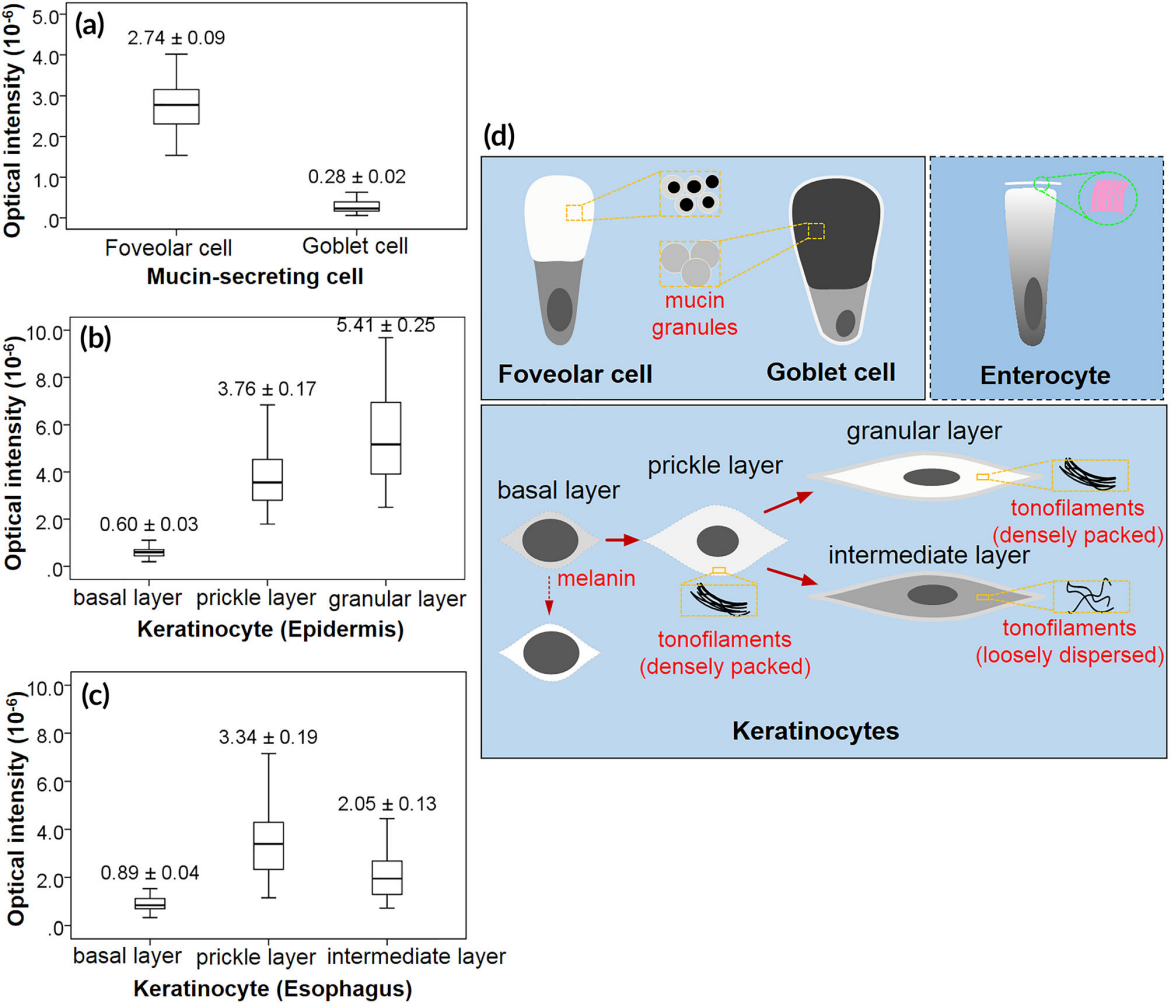
Summary of cytoplasmic reflectance features of specialized epithelial cells. (a–c) Boxplots of cytoplasmic optical intensity of cells in swine epithelial tissues: (a) comparison between foveolar cells and goblet cells; (b) comparison of epidermal keratinocytes from basal, prickle and granular layer, respectively; (c) comparison of esophageal keratinocytes from basal, prickle and granular layer. Data are mean ± *SE*. (d) Schematics of optical reflectance contrast and its ultrastructural feature of epithelial cells under different physiological states

Those cells rich in densely packed tonofilaments presented much higher normalized back‐scattered intensity than those with loosely dispersed tonofilaments (Figure 8b,c). Specifically, in the parakeratinized swine esophageal epithelium, keratinocytes in the prickle layer demonstrated a cytoplasmic intensity of (3.34 ± 0.19) × 10^−6^, which is significantly higher than that of the intermediate layer ([2.05 ± 0.13] × 10^−6^; Figure 8c; one‐way analysis of variation [ANOVA], *p* < .01). These measured data agree with the variations in the packing state of the filaments (Figure 6d,e) and the FDTD predictions (Supporting Information Table 3). Note that in swine epidermis cells at the granular layer was brighter than that at the prickle layer (Figure 8b; one‐way ANOVA, *p* < .01), which is known due to the densely packed keratohyalin granules.^37^

## 4 REFLECTANCE CONTRASTS ON CHARACTERIZING PRECANCEROUS LESIONS

With the establishment of the correlation between the reflectance contrast of epithelial cells and the corresponding cytological and ultrastructural bases (Figure 8d), we attempted to evaluated the feasibility of these improved understandings on characterizing epithelial precancerous lesions.

### 4.1 Simple columnar epithelium

#### 4.1.1 Human gastric mucosa with intestinal metaplasia

**Figure 9:**
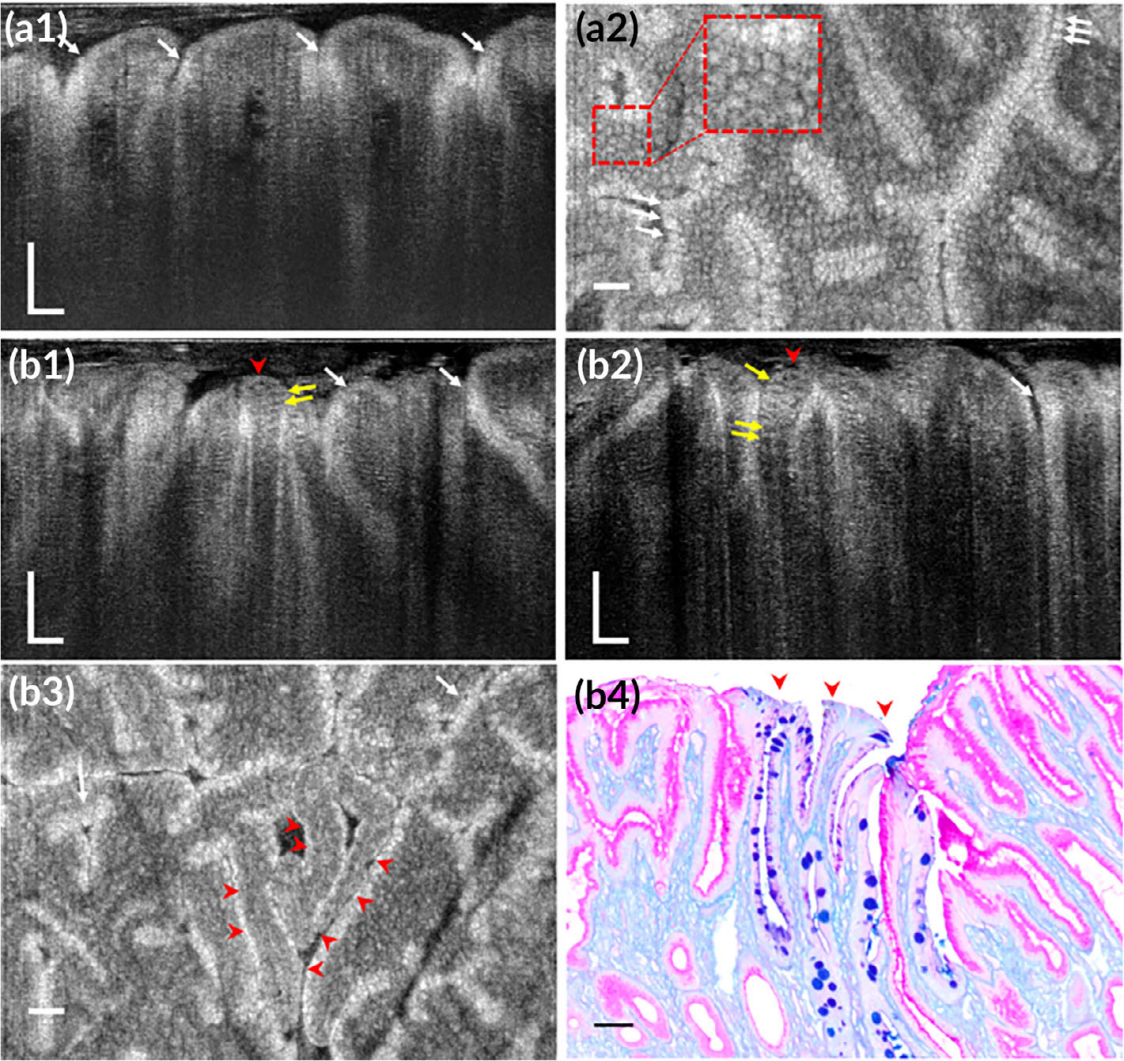
Subcellular reflectance contrast of human gastric mucosa with intestinal metaplasia imaged by μOCT ex vivo. (a1 and a2) OCT cross‐sectional (a1) and en face (a2) images of a specimen with normal mucosa and the white arrows indicates foveolar cells. (b1–b4) OCT cross‐sectional (b1 and b2) and en face images (b3) of another specimen from the region with intestinal metaplasia (red arrowheads): White arrows in (b1–b3) indicate foveolar cells and yellow arrows in (b1 and b2) represent “ectopic” goblet cells featured with low‐scattering apical cytoplasm; corresponding pathology (b4) with PAS‐AB mucin staining to highlight metaplastic area (red arrowheads). Scale bars, 50 μm

In normal gastric mucosa, human foveolar cells which contains exclusively of neutral mucins (only periodic acid‐schiff [PAS] positive) presented high scattering at the apical cytoplasm relative to the basal portion (Figure 9a1–b3, white arrows), allowing the delineation of the mucosal wrinkles and the opening of gastric pits. By contrast, in the region with pathologically diagnosed intestinal metaplasia (IM), metaplasia could be readily differentiated from the normal gastric mucosa as cell clusters were devoid of the characteristic high‐intensity mucin cups of foveolar cells (Figure 9b1–b3, red arrowheads). According to the anatomically matched histology, those cells probably corresponded to the specialized columnar cells whose apical cytoplasm appeared purple with PAS‐alcian blue (PAS‐AB) staining (Figure 9b4). Interspersed between those columnar cells, goblet cells could be detected by their barrel‐ or cylinder‐shaped low‐scattering apical cytoplasm (Figure 9b1,b2, yellow arrows) which were stained blue with PAS‐AB staining (Figure 9b4).

#### 4.1.2 Human colon polyps

**Figure 10:**
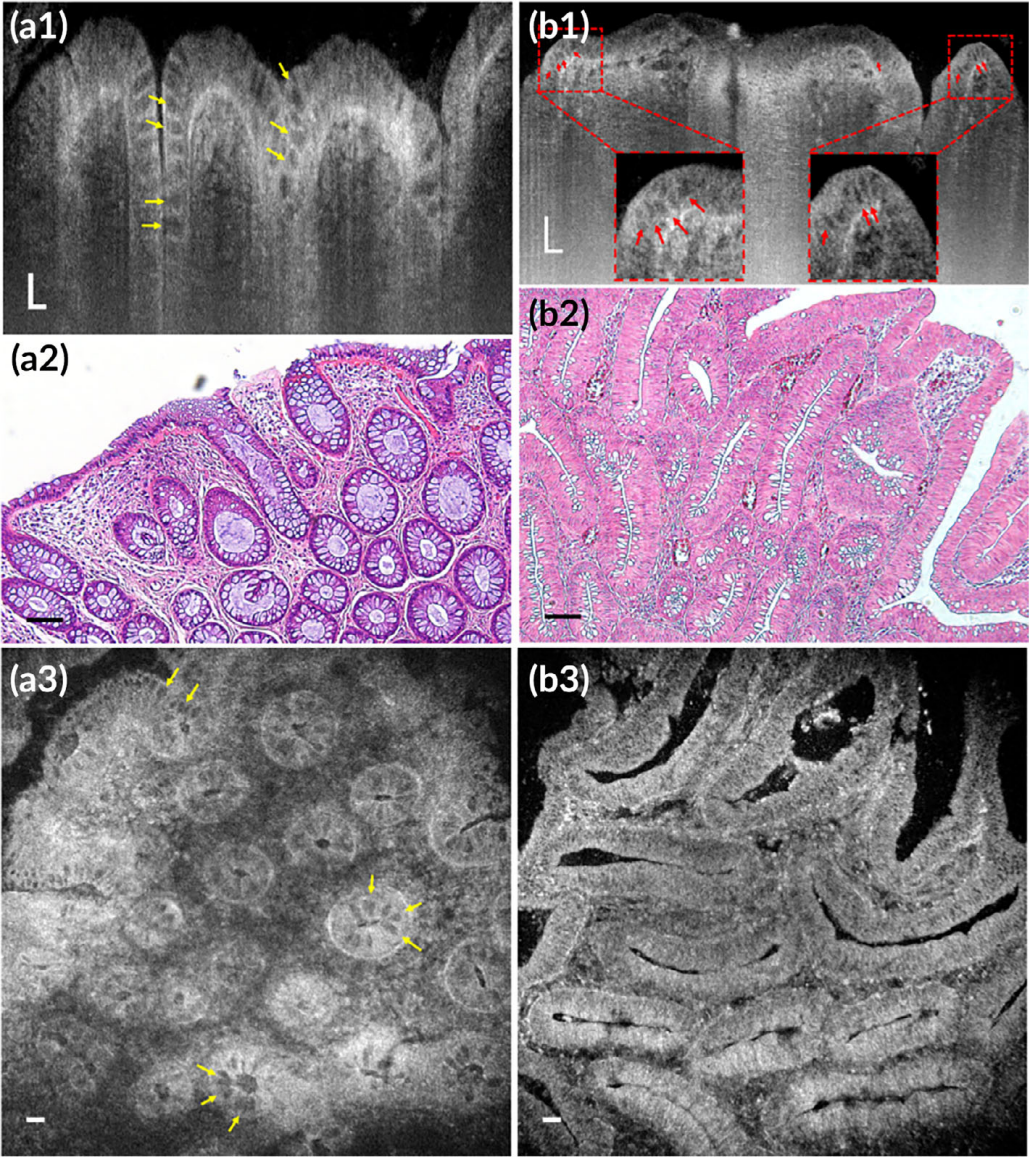
Subcellular optical reflectance contrast of human colorectal polyps imaged by μOCT ex vivo. (a1–a3) Representative μOCT cross‐sectional image (a1), corresponding H&E stained histology (a2), and en face μOCT view (a3) of a hyperplastic polyp. (b1–b3) Representative μOCT cross‐sectional image (b1), corresponding H&E stained histology (b2), and en face μOCT view (b3) of an adenomatous polyp. Yellow arrows represent goblet cells and red arrows indicate low‐scattering nuclei. Scale bars, 50 μm

In non‐neoplastic polyps (Figure 10a2), goblet cells could be readily recognized by their low‐intensity apical cytoplasm relative to the enterocytes (Figure 10a1,a3, yellow arrows). Crypts were typically oval with uniform shape and size manifesting a “honeycomb” pattern and their pits were commonly in small oval shape in the en face view reconstructed from the 3D μOCT dataset (Figure 10a3). Whereas, in μOCT images obtained from polyps with pathologically confirmed adenoma (Figure 10b2), goblet cells were less often visible in comparison with those obtained from non‐neoplastic polyps (Figure 10b1,b3) and dysplastic cells featured with cigar‐shaped pseudostratified nuclei pathologically could be recognized by the elongated, irregularly arranged low‐reflectance nuclei (Figure 10b1, red arrows). Crypts were laterally extended or distorted resulting in large tubular or gyrus‐like pits in the en face view (Figure 10b3). All these μOCT observations matched well with corresponding histology.

### 4.2 Stratified squamous epithelium

#### 4.2.1 Mouse esophagus (orthokeratinized) with intraepithelial neoplasia

**Figure 11:**
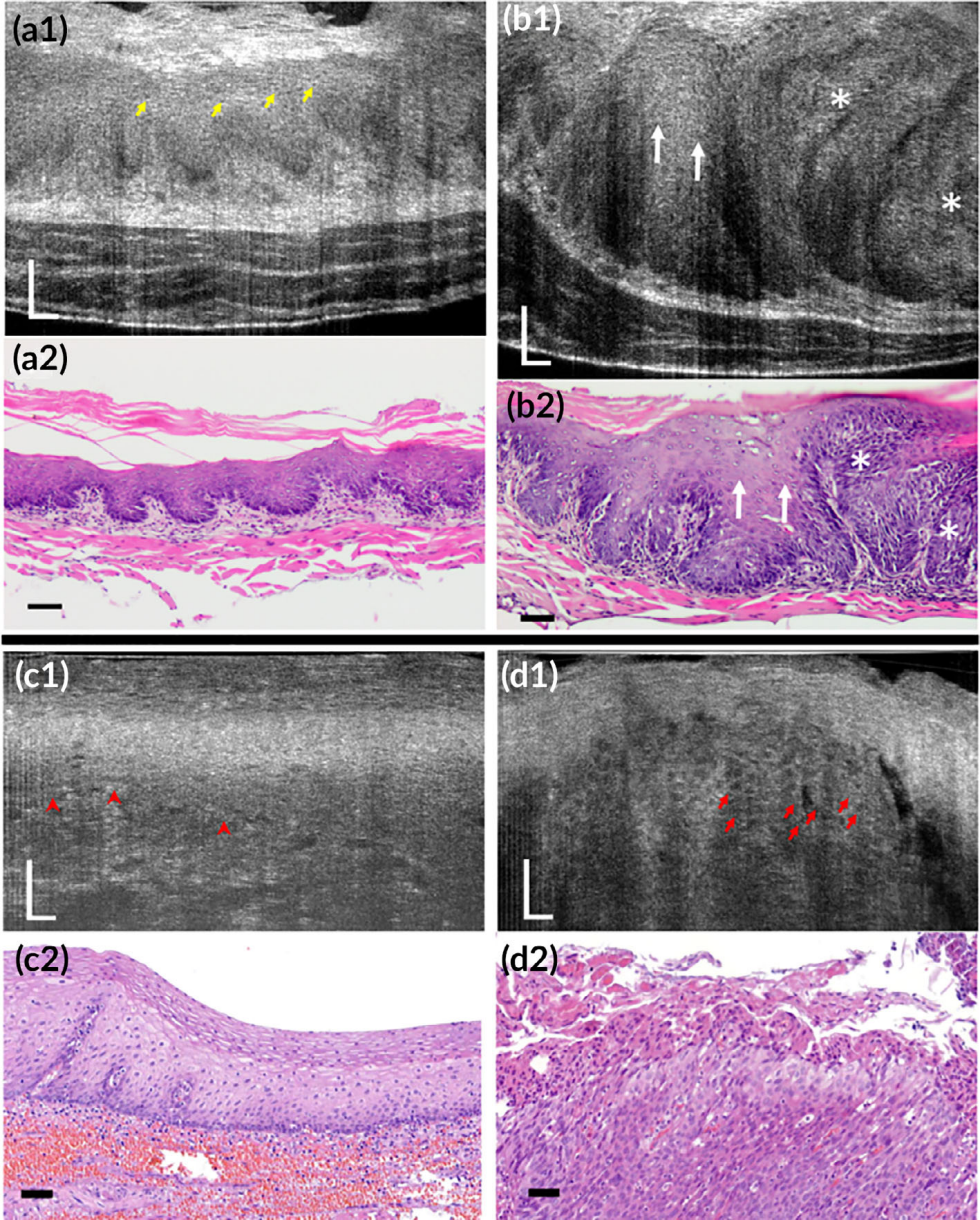
Subcellular reflectance contrast of mouse (a1–b2) and human (c1–d2) esophagus with intraepithelial neoplasia imaged by μOCT ex vivo. (a1, b1, c1, and d1) Representative μOCT cross‐sectional images and (a2, b2, c2, and d2) matched histology with H&E staining: (a1 and a2) a mouse specimen with hyperplasia; (b1 and b2) another mouse specimen with intraepithelial neoplasia where white arrows indicate mild dysplasia and white stars indicate severe dysplasia; (c1–d2) a patient specimen confirmed with severe dysplasia acquired from the clean margin (c1 and c2) and the neoplastic region (d1 and d2): Red arrowheads indicate boundary between epithelium and lamina propria and red arrows suggest low‐scattering nuclei. Scale bars, 50 μm

μOCT images acquired from hyperplastic epithelium resembled the features observed in the normal orthokeratinized epithelium: well‐differentiated keratinocytes demonstrated high‐intensity cytoplasm and detectable nucleocytoplasmic contrast covered by a well‐developed keratinized layer (Figure 11a1,a2). However, images obtained from regions with pathologically confirmed severe dysplasia showed a relatively low optical intensity occupied by immature dysplastic cells with enlarged nuclei and little cytoplasm (Figure 11b1,b2, white stars), in contrast to the neighboring region with mild dysplasia where cells particularly in the upper two‐thirds of the epithelium were well differentiated with rich cytoplasm (Figure 11b1,b2, white arrows).

#### 4.2.2 Human esophagus (nonkeratinized) with intraepithelial neoplasia

In the μOCT images acquired from the Lugol's‐positive clean margin of human esophageal specimen, we could read out characteristic architectural and cytologic information of the normal nonkeratinized epithelium (Figure 11c1,c2). Keratinocytes at the intermediate layer presented relatively low‐intensity cytoplasm similar to those observed in nonkeratinized floor of month (Figure 11c1). By contrast, in the images obtained from Lugol's‐negative area that was confirmed to be severe dysplasia pathologically, the optical intensity differences divided the epithelium into two portions: the surface high‐intensity portion and the underlying low‐intensity portion (Figure 11d1,d2). According to the corresponding histology (Figure 11d2), the high‐intensity portion was consisted of cells with flatted nuclei and rich cytoplasm indicating well cell differentiation and the underlying portion was occupied by cells with oval‐shaped enlarged nuclei suggesting impaired cell maturation.

## 5 DISCUSSION

OCT technology is rapidly advancing toward optimal clinical utilities from bulky bench‐top workstation to compact probe‐ or capsule‐based endomicroscopy, which will open up new avenues for the screening and detection of epithelial disorders.^15^, ^16^, ^17^, ^38^, ^39^, ^40^, ^41^ Improving our understandings of the information encoded in the images is one of the fundamental steps to realize the potentials of this promising technology. The present study uncovers that nanometer‐scale organelles such as mucin granules, keratin filaments and microvilli are significant or main sources of back‐scattered signals, which has never been reported before. The FDTD simulation results reveal the correlations between the back‐scattering contrasts and the ultrastructural clustering or packing states of the organelles, which are validated by the measured data in μOCT images. All these results establish that the reflectance contrasts of μOCT images could be used to decode the physiological/pathophysiological states of epithelial cells, a new step forward toward the long sought goal of “virtual histology.”

Our results for the first time open up the possibility to distinguish different mucin‐secreting cells that are similar in morphology and function in intact tissues without the need of exogenous contrast agents, which has not been proven possible despite the resolving power has been pushed to the cellular level.^14^, ^34^ The study indicates that this advance will reasonably help with the detection of IM in Barret's esophagus and stomach,^42^, ^43^ which is challenging to be targeted with traditional video‐imaging modalities even when a rigorous “systematic biopsy” protocol is used due to inadequate contrast between IM and surrounding epithelium.^5^, ^19^ Besides, as cellular information could be differentiated based on intrinsic contrast, it would possibly eliminate labor‐intensive staining procedures and concerns on the cytotoxicity of contrast agents.^13^, ^14^ In addition, although human foveolar cells contain exclusively of neutral mucins which is different from those of swine, their apical portion also present high optical intensity. This agreement in the back‐scattering features of foveolar cells is possibly owing to that they share a similar ultrastructural feature in the mucin granules.^44^

The characterization of colon polyps exemplifies another clinical benefit to understand the subcellular reflectance contrast. With the expanding implementation of colonoscopy for cancer screening, colorectal polyps are increasingly detected.^12^ However, since more than 90% polyps are small with approximately 50% of them being non‐neoplastic,^12^, ^45^, ^46^ pathologic assessment is in most cases used only to determine surveillance intervals,^12^, ^46^ which give rise to significant healthcare costs.^12^, ^47^ Based on the understanding of reflectance contrasts established in this study, we show that pathology hallmarks like decreased goblet cells, pseudostratified nuclei and irregular crypt patterns in adenomatous polyps can be well recognized in μOCT images. This capability offers the possibility to differentiate adenomatous polyps from non‐neoplastic polys in real time, which would possibly save substantial pathology costs in colon cancer prevention.^12^, ^47^

The current study also provides us a comprehensive understanding of the reflectance signals from nonpigmented keratinocytes. We disclosed that the cytoplasmic reflectance intensity was correlated with the packing state of keratin filaments. With the biological bases underlying subcellular reflectance contrast of keratinocytes clarified, we demonstrate the capability to distinguish between differentiated cells and dysplastic cells in both the orthokeratinized and nonkeratinized SSEs. Since the packing state of keratin filament is associated with the level of keratinization, this knowledge may also be useful for grading cancer cells in the squamous cell carcinoma because the synthesis as well as the packing state of keratin filaments varied between well‐differentiated and poorly‐differentiated neoplastic cells.^48^ Additionally, the reflectance contrast difference between the squamous and columnar epithelium at the squamocolumnar junction suggests a possibility to detect squamous metaplasia in the simple columnar epithelium, which is a preneoplastic change in organs like cervix and lung.^49^

While few tools are available in optimally evaluating brush border except for histology, we demonstrate the possibility to evaluate this polarization‐derived subcellular structure using μOCT noninvasively and in real‐time. This ability may benefit the research on a number of microvilli related abnormalities, such as celiac disease^50^ and microvillus inclusion disease.^51^

Back‐scattered contrast depends on complex processes of Mie and Rayleigh scattering, involving relative refractive indices, shape and orientation and clustering of structures, etc. The contrast seen in optical reflectance images is due to an ensemble of scattering events—those in the optical section that are modulated by those in the tissue layers above and below. Our numerical models were developed based on multiple assumptions in the shape, orientation, distribution, and refractive index of the clustered and packed scatterers. These simplification strategies are necessary since some of these data is not available or measurable. In our study, this shortcoming is compensated by the experimental validations. However, our numerical modeling cannot include all the biological variations of the scatterers under investigation, such as keratin filaments in the keratinized layers and basal layers of the stratified squamous epithelium. In these cases, speculative observations are reported based on our experiences.

Our preliminary study provided promising results on distinguishing precursor lesions from normal mucosa but future multicenter studies are necessary to validate the efficiency and accuracy of these criteria. Although demonstrated with μOCT, these understandings on the biological meaning of reflectance contrast should be directly applicable to other optical reflectance techniques including but not limited to RCM,^13^, ^33^ OCM,^34^ and FF‐OCT.^52^ Looking forward, with the rapid advances in the development of cellular‐resolution optical reflectance technologies,^17^, ^18^, ^53^, ^54^ diagnostic criteria based on these new understandings would complement the current morphology‐ or architecture‐based criteria to specifically decode the pathophysiological events within tissues. The success in this effort would ultimately have a substantial impact for the screening and surveillance of epithelial diseases by reducing costs and risks from unnecessary resections and sampling errors.

## 6 MATERIALS AND METHODS

### 6.1 OCT system

We used a μOCT system described in one of our previous works.^23^ The system provides a transverse resolution of 1.8 μm and an axial resolution of 1.28 μm in tissue (refractive index = 1.33). The axial focus, defined as the axial range of 6‐dB intensity roll‐off, was ~115 μm and the depth of focus was 32.6 μm. The data size of each three‐dimensional (3D) dataset was 1,024 × 1,024 × 2,048 (*x* × *y* × *z*), and the corresponding scanned volume was 0.872 mm × 0.872 mm × 0.88 mm (width × height × depth), in which the depth was measured in water (refractive index = 1.33). An axial line rate of 20 and 60 kHz was used for ex vivo and in vivo studies, respectively.

### 6.2 Modeling of scatterers using FDTD method

Modeling of clustered or packed nanometer scale scatterers was conducted using Lumerical software (Lumerical FDTD Solutions, Vancouver, Canada). We followed the method of modeling reported in our previous study.^55^ Simulating the reflectance imaging system of μOCT was conducted using MatLAB (MathWorks, MA) based on the scalar diffraction theory and the optical parameters of the sample arm optics, which are described in detail previously.^55^ The spatial and optical parameters of models were obtained from TEM images and published data, and are provided in the Supporting Information Tables 1–3.

### 6.3 Normalized back‐scattering intensity calculation and measurement

The normalized back‐scattered intensity is defined as the ratio between the back‐scattered light intensity from the scatterers under investigation and the back‐reflected intensity from a gold‐coated mirror. In μOCT‐based measurements, we firstly measured the back‐reflected intensity from a gold‐coated mirror, and then normalize all the back‐scattered intensity to it. By doing so, the measured values are independent of the imaging system, and therefore, can be compared between different experiments and with the simulated values.

### 6.4 Transmission electron microscopy

Specimens harvested from anesthetized cross Landrace swine (female, 12 months) were immediately immersed in 3% glutaraldehyde (pH = 7.4) at 4°C for prefixation. After being trimmed to ~2 × 1 × 1 mm, they were fixed with fresh 3% glutaraldehyde (pH = 7.4) for ~6 hr and postfixed with 1% osmium tetroxide for ~2 hr at 4°C. Following routine TEM procedures, the specimens were embedded with Epon812. After the successful identification of intact mucosa by semithin sectioning, specimens were sectioned to 70‐nm by ultramicrotome (LKB Ultrotome V) and stained with uranyl acetate and lead citrate. The ultrastructural characteristics of epithelial cells from different organs were observed under TEM (JEOL 1200EX) and recorded for analysis with Olympus Morada G2 camera.

### 6.5 Swine epithelial tissues

Skin, floor of mouth, esophagus, stomach, small intestine, and colon from four cross Landrace white pigs (female, 12 months) were harvested immediately after the cessation of vital signs. Specimens with a size of 1–2 cm × 2 cm were separately stored in serum‐free Dulbecco's modified Eagle's medium (DMEM; Gibco). Specimens were shipped on ice within 1 hr to the imaging site, where they were preserved in DMEM at 37°C ventilated with 95% oxygen and 5% carbon dioxide. μOCT image acquisition of all specimens was completed within the next 1 hr. Following μOCT imaging, the specimens were submitted for routine histological procedures, sectioned into 5‐μm sections (RM2235, Leica Biosystems), and stained with hematoxylin–eosin (H&E; Leica Biosystems). To evaluate the histochemical property of mucins between mucus‐secreting cells, we also performed PAS and AB staining of sections from stomach and colon (Leica Biosystems). The use of swine tissues was approved by the Institutional Animal Care and Use Committee (IACUC) of National University of Singapore (R14‐0797).

We also conducted μOCT imaging of above‐mentioned tissues except for the oral mucosa in another two white micropigs (female, 12 months) in vivo. We anesthetized the animals and surgically opened the lumen to access the epithelial surface of internal organs. Epithelial surface of each organ was washed with phosphate buffered saline (pH = 7.4; Gibco) prior to μOCT image acquisition. The study was approved by the IACUC of PWG Genetics Pte, Ltd. (PN16076).

### 6.6 Rat cervical and vaginal epithelium

A total of 14 Sprague Dawley rats (female, 10–14 weeks old) were used in this study with two for cervical imaging in vivo and 12 for vaginal imaging ex vivo. The estrous stage was evaluated by the features of exfoliated vaginal cells prior to μOCT image acquisition. Specifically, the rats were housed under a standard 12:12 light–dark cycle, and vaginal cell samples were collected at 10:00 a.m. every day using the pipette smear technique.^56^ Following sample collection and smearing, the air‐dried cells were stained with 0.1% crystal violet solution (Sigma‐Aldrich) for 1 min, washed twice in ddH_2_O to remove excess stain and examined under a light microscope (Olympus BX53).

For cervical study in vivo, rats at the proestrus were anesthetized and we performed μOCT imaging after surgical exposure of the squamocolumnar junction. For vaginal study ex vivo, rat was sacrificed and μOCT images were acquired from the luminal side of the upper third of the vagina within 30 mins after tissue harvest. Following image acquisition, regions of interest were fixed with 10% neutral‐buffered formalin (Leica Biosystems) and 5 μm‐thick sections were stained with H&E for histological analysis. These studies were approved by IACUC of NTU (ARF‐SBS/NIE‐A0312).

### 6.7 Mouse esophagus and human specimens with epithelial precancerous lesions

C57BL/6 mice (female, 6 weeks) treated with 4‐nitroquinoline 1‐oxide (4NQO) at 100 μg/mL in the drinking water were used to develop esophageal cancer.^31^ The mice were sacrificed every 2 weeks starting in week 14 and the esophagus was harvested, opened longitudinally. μOCT images were sequentially acquired from 6–10 locations from the proximal to the distal end. Regions with visible tumors or intratumor vessels were excluded. Following OCT imaging, specimens were fixed for pathological analysis. OCT datasets with landmarks such as blood vessels and epithelial rete pegs that well‐matched with the corresponding histopathology were selected for analysis. We acquired approval from the IACUC of NTU (ARF‐SBS/NIE‐A0319).

We also obtained images from human endoscopically resected specimens. Four esophageal specimens with pathologically confirmed severe dysplasia or superficial cancer were used and μOCT images were acquired from both the Lugol's‐positive margin and the Lugol's‐negative region (Supporting Information Figure 4a). In stomach, three specimens were included including two without epithelial lesions and one with IM. In the one with IM, μOCT images were obtained from the smaller depressed lesion which were confirmed with monofocal IM pathologically (Supporting Information Figure 4b; red arrow). Eleven colorectal polyps including six nonadenomatous polyps and five adenomatous polyps were included, and μOCT images were acquired from 2 to 4 random locations for each specimen. Following image acquisition, all the specimens were fixed overnight and subject to a standard surficial specimen processing protocol. The specimen was sectioned at the interval of 2 mm in width and embedded in its entirety for histopathological diagnosis. Written informed consent was obtained from participants prior to the study and the use of human tissues was approved by the IRB at Renmin Hospital of Wuhan University (2017K‐C053).

### 6.8 Statistical analysis

A quantitative analysis of the cytoplasmic optical intensity were conducted among cells in normal swine epithelial tissues. We manually measured the averaging intensity from 50 randomly selected locations within an area of 10 × 8 pixels (width × height) for each location. The data for each epithelial type were from the 1,024 images of one 3D OCT dataset. Independent‐samples *t* test and one‐way ANOVA was adopted to compare optical intensity in‐between mucus‐secreting cells and among keratinocytes at different level of maturation, respectively. Further Bonferroni post hoc test was performed if statistical differences were detected. A *p* < .05 was regarded as a statistically significant difference. All numerical values were presented as mean ± *SE*. All statistical analyses were conducted using SPSS software (IBM SPSS Statistics 23.0).

## CONFLICT OF INTEREST

The authors have no conflicts of interest to declare.

## Supporting information


**Data S1** Supporting information.
