# Supplementary material for: Understanding optical reflectance contrast for real‐time characterization of epithelial precursor lesions
Source: Bioeng Transl Med. 2019 Jun 27;4(3):e10137. doi: 10.1002/btm2.10137 (PMC6764805; doi:10.1002/btm2.10137)
Supplement: Supplementary file 1 — Data S1 Supporting information. [file BTM2-4-na-s001.docx]

**APPENDIX**

**Supplementary Table 1**. Parameters of mucin granules in FDTD modelling

| **Optical parameters** | **Value** | |
| --- | --- | --- |
| Sources | Gaussian wave | |
| Collection numerical aperture | 0.2 (1% power level) | |
| Wavelength (nm) | 700-950 with a 6.25 increment | |
| Mesh grid (nm) | 30 | |
| Polarization | Unpolarized | |
| Refractive index of cytosol | 1.34[1] | |
| **Mucin granules** |  | |
| Refractive index | 1.38[2] | 1.41[2] |
| Diameter (nm) | 202 (137-265)^a^ | 202 (137-265)^a^ |
| Density (numbers/μm^3^) | 18.1^b^ | 18.1^b^ |
| Focal volume  (μm (x) × μm (y ) × μm ( z)) | 2 × 2 × 2 | 2 × 2 × 2 |
| **Normalized back-scattered intensity** | 6.7 × 10^-6^ | 1.8 × 10^-5^ |

^a^Data acquired from TEM images of swine stomach (n=50).

^b^Determined by the center-center inter-granules distance of ~380 nm measured from TEM images of swine stomach (n=50).

**Supplementary Table 2.** Parameters of microvilli in FDTD modelling

| **Optical parameters** | **Value** | | | |
| --- | --- | --- | --- | --- |
| Sources | Gaussian wave | | | |
| Collection numerical aperture | 0.2 (1% power level) | | | |
| Wavelength (nm) | 700-950 with a 6.25 increment | | | |
| Mesh grid (nm) | 30 | | | |
| Polarization | Unpolarized | | | |
| Refractive index of cytosol | 1.34[1] | | | |
| **Microvilli** | **Small intestine** | | **Colon** | |
| Refractive index | 1.38[2] | 1.41[2] | 1.38[2] | 1.41[2] |
| Diameter (nm) | 100[3] | | 90 | |
| Height (nm) | 1010-1172[3] ^a^ | | 140 - 510^b^ | |
| Density (numbers/μm^2^) | 81.8[3] | | 33.0^c^ | |
| Focal volume  (μm (x) × μm (y ) × μm ( z)) | 2 × 2 × 2 | | 2 × 2 × 2 | |
| **Normalized back-scattered intensity** | 2.5 × 10^-5^ | 7.4 × 10^-5^ | 0.39 × 10^-5^ | 1.2 × 10^-5^ |

^a^ Measured over 2-µm lateral field of view.

^b^ Data acquired from TEM images of swine colon (n=50) over 2-µm lateral field of view.

^c^ Determined by the center-center inter-microvillus distance of 140-340 nm (n=50).

**Supplementary Table 3.** Parameters of keratinocyte filament in FDTD modelling

| **Optical parameters** | **Value** | |
| --- | --- | --- |
| Sources | Gaussian wave | |
| Collection numerical aperture | 0.2 (1% power level) | |
| Wavelength (nm) | 700-950 with a 6.25 increment | |
| Mesh grid (nm) | 30 | |
| Polarization | Unpolarized | |
| Refractive index of cytosol | 1.34[1] | |
| **Keratin filament type** | **Densely packed tonofilament bundles** | **Loosely dispersed individual tonofilaments** |
| Packing state | parallel and dense packing | parallel and loose packing |
| Orientation | Perpendicular to the light propagation | Perpendicular to the light propagation |
| Refractive index | 1.40[1] | 1.40[1] |
| Diameter (nm) | 8 | 8 |
| Density (numbers/μm^2^) | 5972^a^ | 4276^b^ |
| Structure volume  (μm (x) × μm (y) × μm (z)) | 0.5 × 0.5 × 0.5 | 0.5 × 0.5 × 0.5 |
| Focal volume  (μm (x) × μm (y ) × μm ( z)) | 2 × 2 × 2 | 2 × 2 × 2 |
| Number of structures per focal volume | 14^c^ | 14^c^ |
| **Normalized back-scattered intensity** | 5.46 × 10^-6^ | 3.08 × 10^-6^ |

^a,b^Determined by the center-center inter-tonofilament distance of 16 nm and 24 nm, respectively.

^c^Estimated based o TEM images of swine epidermis.

**References**

1. S. A. Leachman, "Methods of Melanoma Detection," in *Melanoma*, J. M. M. Howard L. Kaufman, ed. (Springer International Publishing, 2016), p. 79.

2. H. Liu, B. Beauvoit, M. Kimura, and B. Chance, *Dependence of tissue optical properties on solute-induced changes in refractive index and osmolarity* (SPIE, 1996), Vol. 1, pp. 200-211, 212.

3. J. A. Pinette, S. Mao, B. A. Millis, E. S. Krystofiak, J. J. Faust, and M. J. Tyska, "Brush border protocadherin CDHR2 promotes the elongation and maximized packing of microvilli in vivo," Molecular biology of the cell **30**, 108-118 (2019).

**Supplementary Figures**


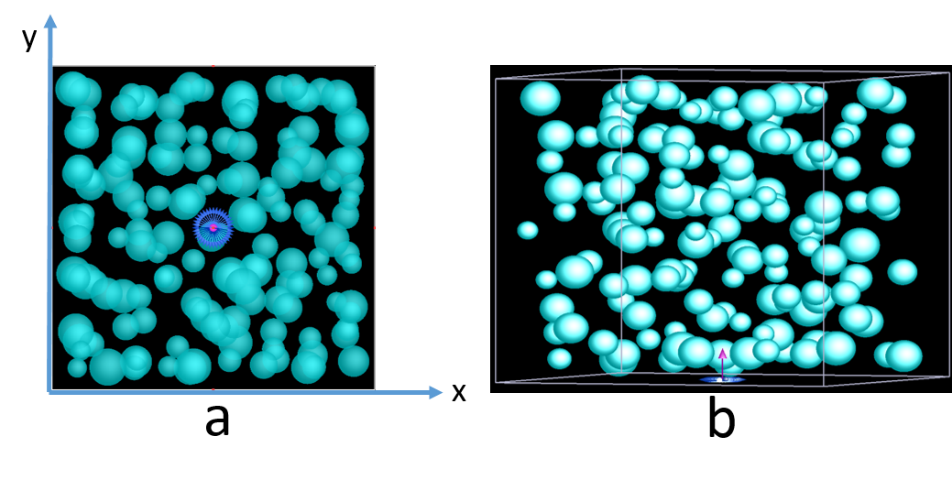


**Supplementary Figure 1.** Numerical model of clustered mucin granules in swine foveolar cells. (a) *en face* view; (b) Three-dimensional view. Size of the volume: 2 µm × 2 µm × 2 µm.


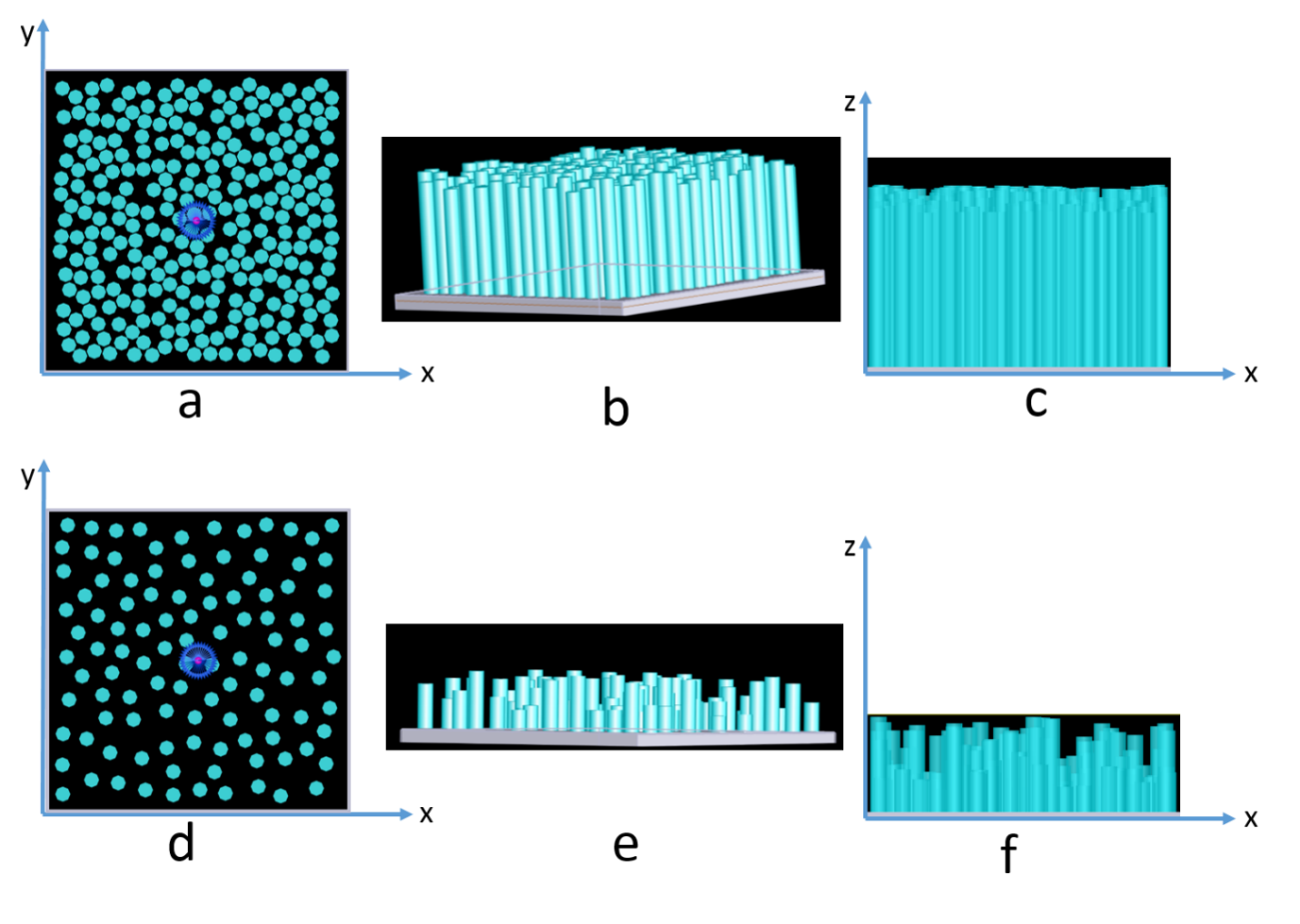


**Supplementary Figure 2.** Numerical models of microvilli. (a-c) brush border in the enterocytes of the small intestinal: *en face* view (a), three-dimensional view (b), and side view (c); (d-f) microvilli in the colon epithelial cells: *en face* view (d), three-dimensional view (d), and side view (f). Size of the volume: 2 µm × 2 µm × 2 µm.


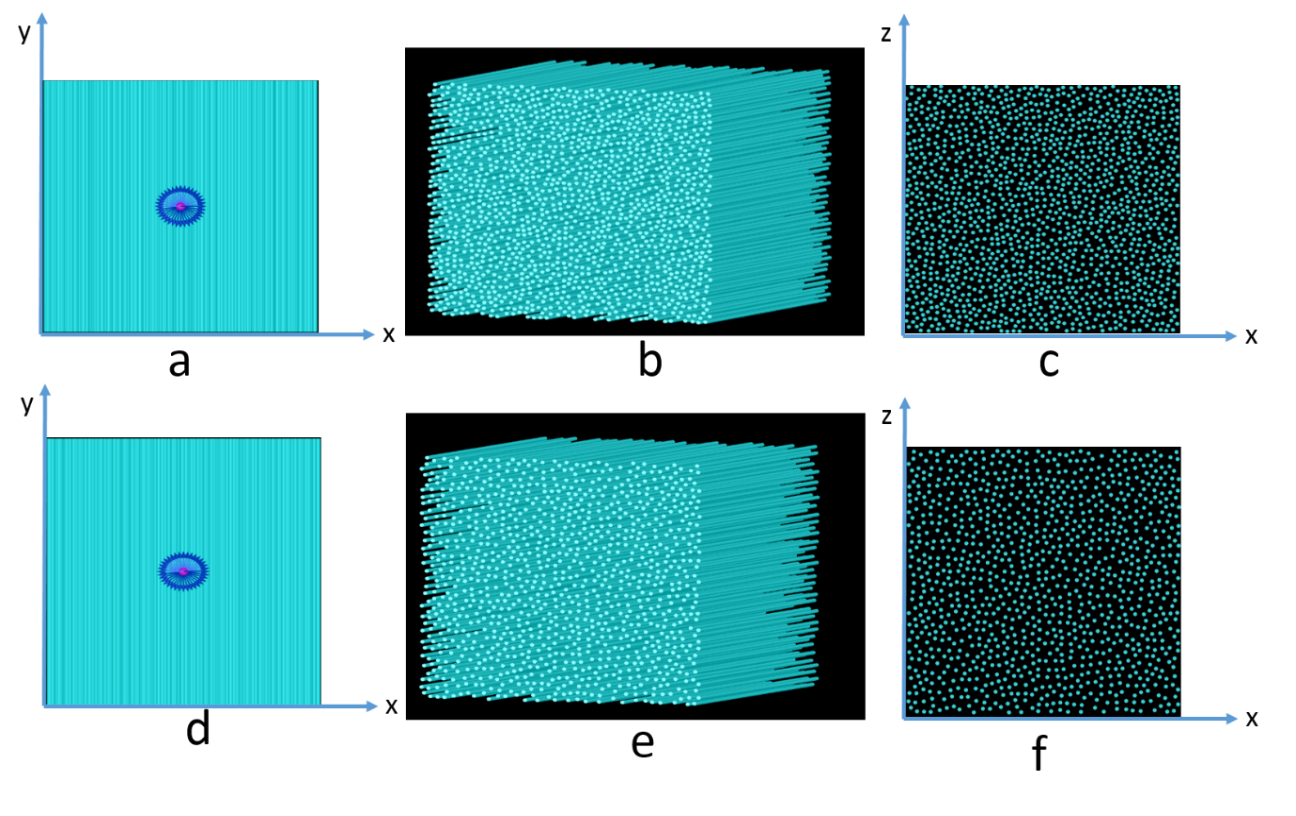


**Supplementary Figure 3.** Numerical models of keratin filaments. (a-c) a densely packed tonofilament bundle: *en face* view (a), three-dimensional view (b), and side view (c); (d-f) loosely packed keratin fibers cells: *en face* view (d), three-dimensional view (d), and side view (f); Size of the volume: 0.5 µm × 0.5 µm × 0.5 µm.


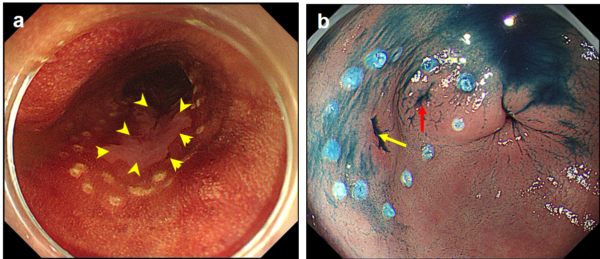


**Supplementary Figure 4. Endoscopic photographs of human esophagus and stomach.** (a) A patient’s esophagus (female, 59 years old) with Lugol’s solution staining: the Lugol’s negative area (yellow arrows) is confirmed with severe dysplasia pathologically and the rest normal mucosa is stained brown. (b) A patient’s stomach (male, 52 years old) with indigo carmine staining: the smaller depressed lesion (red arrow) is confirmed with intestinal metaplasia pathologically and the other depressed lesion (yellow arrow) is introduced by preoperative biopsy.
